# Online interventions for depression and anxiety – a systematic review

**DOI:** 10.1080/21642850.2014.945934

**Published:** 2014-08-20

**Authors:** Sahoo Saddichha, Majid Al-Desouki, Alsagob Lamia, Isabelle A. Linden, Michael Krausz

**Affiliations:** ^a^ Deparment of Psychiatry, Melbourne Health, Melbourne, VIC3000, Australia; ^b^ Department of Psychiatry, King Khalid University Hospital, Riyadh, Saudi Arabia; ^c^ Faculty of Medicine, King Saud bin Abdul Aziz University for Health Sciences, Riyadh, Saudi Arabia; ^d^ Department of Psychiatry, University of British Columbia, Vancouver, Canada

**Keywords:** online CBT, Web-based interventions, anxiety, depression

## Abstract

*Background*: Access to mental health care is limited. Internet-based interventions (IBIs) may help bridge that gap by improving access especially for those who are unable to receive expert care. *Aim*: This review explores current research on the effectiveness of IBIs for depression and anxiety. *Results*: For depression, therapist-guided cognitive behavioral therapy (CBT) had larger effect sizes consistently across studies, ranging from 0.6 to 1.9; while stand-alone CBT (without therapist guidance) had a more modest effect size of 0.3–0.7. Even other interventions for depression (non-CBT/non-randomized controlled trial (RCT)) showed modestly high effect sizes (0.2–1.7). For anxiety disorders, studies showed robust effect sizes for therapist-assisted interventions with effect sizes of 0.7–1.7 (efficacy similar to face-to-face CBT) and stand-alone CBT studies also showed large effect sizes (0.6–1.7). Non-CBT/Non-RCT studies (only 3) also showed significant reduction in anxiety scores at the end of the interventions. *Conclusion*: IBIs for anxiety and depression appear to be effective in reducing symptomatology for both depression and anxiety, which were enhanced by the guidance of a therapist. Further research is needed to identify various predictive factors and the extent to which stand-alone Internet therapies may be effective in the future as well as effects for different patient populations.

## Introduction

Although there is an increasing recognition of mental health issues around the world, accessibility to healthcare has been a key problem, with specialist access in psychiatry restricted to only about 10% (Wang et al., 2005). In fact, less than a third of all patients get access to basic care (e.g. seeing a primary-care physician), and the majority (two-thirds) receives no access at all (Wang et al., 2005). Developing appropriate support strategies for the vast majority, especially for highly prevalent problems, such as mood disorders, anxiety disorders and substance-use disorders, is a critical public health challenge. Online interventions have the potential to address this gap for a variety of disorders and problems, including substance abuse, depression, anxiety, lack of social skills and panic disorders (Barak, Hen, Boniel-Nissim, & Shapira, 2008; Marks, Cavanagh, & Gega, 2007). Delivery of interventions through the Internet provides anonymity and easy accessibility, therefore making it a suitable option for clients with psychological problems to receive help. In addition, they can avoid the stigma incurred by seeing a therapist (Gega, Marks, & Mataix-Cols, 2004), and can obtain treatment at any time or place, work at their own pace, and review the material as often as desired.

With Internet-based interventions (IBIs), clients can be supported in a variety of different ways, from screening to structured assessments, and from guided self-help to sophisticated expert-system-based treatments. The level of therapist involvement can vary from no assistance or minimal therapist contact by e-mail or telephone, to the amount of involvement as seen in classic individual therapy. Thus, it may be possible to reduce the therapist involvement time while maintaining efficacy (Wright et al., 2005). Furthermore, it may be possible to reach people through the Internet who might not otherwise receive treatment for their conditions. These advantages outline the popular support that online interventions have received. Yet, the effectiveness of online interventions still needs to be evaluated in order to gain a clear understanding of their potential cost–benefit ratio.

In the past, there have been some excellent systematic and meta-analytical reviews evaluating online interventions. Griffiths and colleagues (Griffiths, Farrer, & Christensen, 2010) evaluated 26 randomized controlled trials (RCTs) on depression and anxiety interventions, excluding all other kinds of studies. They found an effect size difference ranging from 0.42 to 0.65 for depression interventions and 0.29–1.74 for anxiety interventions. Another review which evaluated online interventions for depression and anxiety concentrated on children and adolescents (Calear & Christensen, 2010). This paper compared four intervention programs describing eight different studies. Although it described each of the programs, it did not evaluate for effectiveness. An earlier meta-analysis of 12 RCTs in 2007 (Spek, Cuijpers et al., 2007) evaluated online interventions for depression and anxiety through a mixed effects analysis and found small mean effect sizes for depression (0.32) compared to larger ones for anxiety (0.96). Therapist-supported interventions also had larger effect sizes than the non-therapist ones.

Another quantitative review (Reger & Gahm, 2009) which combined computer-based and Internet-based cognitive behavioral therapy (CBTs) (ICTs) evaluated 19 RCTs and observed ICT was superior to wait-list and placebo assignment across outcome measures (ds = 0.49–1.14), with effects of ICT being equal to therapist-delivered treatment across anxiety disorders. Titov (2011), in his systematic review of 13 studies that dealt with Internet delivered interventions for depression, observed therapist-guided Internet cognitive behavioral therapy (ICBT) to be as effective as face–to-face psychotherapy, although there were no direct comparisons of effect sizes made across studies. A similar meta-analysis of 12 randomized trials of online interventions (which also included two computer-based studies) for depression observed a modest effect size (*d* = 0.41), with supported CBT having larger effect sizes than unsupported ones (Andersson & Cuijpers, 2009). There have been other reviews which have however been excluded since they focused on only computer-based CBT as opposed to Internet-based CBT or a combination of the two. Since most of the earlier reviews mentioned above were rigid in their definitions of the studies being included, several excellent studies remained excluded. This paper therefore systematically reviewed the existing research on online interventions for depression and anxiety disorders by expanding the scope of the search and including all studies on Internet-delivered interventions. It aimed to qualitatively as well as quantitatively review the efficacy of these interventions.

## Method

Articles of potential relevance were identified using PsychInfo and PubMed to search a database of English language abstracts for articles published prior to January 2014. The search was carried out using the keywords “depression”, “anxiety”, “online”, “Internet”, “Web” and combinations thereof. The bibliographies of the articles identified via searches revealed additional sources. Studies were included only if they: (i) involved a self-help website intervention or an online intervention that incorporated a self-help component; (ii) described the website application as targeting a depression or anxiety condition; (iii) tested the efficacy or effectiveness of the intervention; (iv) incorporated a measure of symptom outcome for the targeted condition; and (v) had been peer reviewed and published. We specifically excluded computer-based CBT or tele-psychology, as these do not pertain to a direct online intervention. We also excluded studies, for which complete data were unavailable, which were not in English, or which did not primarily target depression/anxiety. Two independent reviewers reviewed all literature for available interventions and existing research, which was overseen by a senior professor independently. Within-group effect sizes were extracted from either the results section of the individual papers or from the data available using the RevMan 5.0 Meta-analysis calculator (Review Manager 5 (RevMan), 2011). Effect sizes were expressed as Cohen's *d* values.

Although not a primary aim, the methodological quality of the studies was additionally assessed using three basic criteria: (1) clients did not have prior knowledge of treatment assignment; (2) assessors of outcomes were blinded toward treatment assignment; and (3) complete follow-up data were available (Higgins & Green, 2005).

## Results

The exhaustive search yielded more than 840 articles. Abstracts that did not have an online component, described only the program and not the effectiveness of the intervention, or were duplicates were rejected. A total of 43 publications met the inclusion criteria and which reported the results of trials for IBIs were included in Tables 1–5. However, due to the variation in the methodologies of the studies reviewed, such as participant and control group characteristics, the type of intervention delivered, the differing durations of follow ups, it was not possible to compare effect sizes across studies. The studies were therefore systematically reviewed and grouped by treatment target into two categories and presented in Tables 1–5: (a) depression and (b) anxiety disorders. For each, information is provided regarding sample characteristics, intervention conditions, sessions/modules, level of clinician involvement, follow-up periods, and within-group effect sizes.

**Table 1.** RCTs in depression with therapist guidance.

Study	Sample	Intervention	Results	Comments
Choi et al. (2012)	Chinese Australians with DSM IV depression above 18 years. Treatment grp (*n* = 25) vs. wait-listed grp (*n* = 30). Administered the BDI, PHQ 9, DASS 21, Kessler 10 (k 10) psychological distress scale and Sheehan disability scales (SDS) at baseline and 3 months follow-up	Six online lessons on behavioral activation, cognitive restructuring, problem solving, and assertiveness skills; a homework assignment for each lesson; regular automatic reminder and notification e-mails; weekly telephone contact and secure e-mail with Chinese-speaking support personnel	Large between-group effect sizes were found on the CBDI (0.93, CI 95% = −4.57 to 1.88), and moderate between-group effect sizes were found on the CB-PHQ-9 (0.50). 3 months follow-up present	Telephone support present
Kessler et al. (2009)	297 patients aged 18–75 years from primary care with a new episode of depression (score of 14 or more with the Beck depression inventory (BDI) and ICD 10 diagnosis). Randomized to receive online CBT with a therapist or wait control group. Reassessed with BDI and EuroQOl at baseline, 4 and 8 months of treatment	The intervention comprised up to 10 sessions, each of 55 min of CBT delivered online, to be completed within 16 weeks of randomization when possible	BDI and EuroQol scores reduced significantly in the treated group both at 4 months and 8 months. There was also greater recovery from depression in the Intervention group. Follow-up at 4 month and 8 months of treatment	No co-morbidies allowed.
				More severe depression patients in the sample, which is a strength of the study
Vernmark et al. (2010)	88 persons with mild to moderate depression diagnosed using SCID were randomized into three groups, Web-based self-help, E-mail-based therapy, or Wait-listed. All patients were assessed using Beck Depression Inventory (BDI), Montgomery Åsberg Depression Rating Scale-Self-Rated (MADRS-S), Beck Anxiety Inventory (BAI) and the Quality of Life Inventory (QOLI) at baseline, post-treatment, and 6 months follow-up	Guided self-help had seven modules consisting of an introduction to CBT, CBT-perspective with a behavioral focus, behavioral activation, cognitive restructuring, and sleep management, defining goals/values and relapse prevention	Patients in both treatment groups significantly improved at end point (*p* = 0.002 and *p* = 0.06). At six months follow-up, there were no differences between the two treatment groups. Follow-up at 6 months	Small sample size did not permit analysis of inter-group differences
		E-mail therapy comprised individualized treatment protocols based on CBT-principles for treating depression, with a focus on case conceptualization, functional analysis, and subsequent applications of components commonly used in CBT for depression including behavior activation and cognitive restructuring		
Perini et al. (2009)	Forty-five individuals with DSM IV depressive disorder were randomized into either the treatment group (Sadness Program) or wait-listed. The PHQ-9, the Beck Depression Inventory II (BDI-II), the Positive and Negative Affect Scales (PANAS), Kessler 10 (K-10) and the SDS	The Sadness program consists of four components: six online lessons, homework assignments, participation in an online discussion forum, and regular e-mail contact with a mental health clinician	Large between-group effect sizes were found for the PHQ-9 (0.89) and BDI-II (0.63). Others were non-significant	Small sample size
Ruwaard et al. (2009)	39 individuals in the Netherlands with chronic, moderate depression (Score 10–29 on BDI IA), were randomized to receive Web-based CBT or wait-listed. Assessed using Depression Anxiety Stress Scales (DASS-42), BDI-IA, Well-being Questionnaire (WBQ12), SCL-90-R Depression Scale at pre-, post-treatment and 18 months follow-up	The Web-based CBT had eight phases; Awareness by writing; Monitoring mood; Structuring activities; Cognitive restructuring; behavioral experiments; Positive self-verbalization; Social skills training; and Relapse prevention. It was therapist guided	Between-group effect sizes were robust for SCL 90R (1.1), BDI IA (0.7), DASS anxiety (1.0) and WBQ 12 (1.0), which remained robust even at follow-up (1.1–1.6). Follow-up at 18 months	No clear criteria for Chronic depression.
				Severe cases excluded.
				Other co-morbidity excluded
Hollandare et al. (2011)	84 participants with partially remitted major depression (Between 7 and 19 on the Montgomery-Asberg Depression Rating Scale (MADRS-S)) after treatment were randomly assigned to either 10 weeks of Internet-based CBT or to a control group. Primary outcome was relapse to DSM IV depression. Other assessments were MADRS, BDI II, BAI, WHOQOL-BREF	ICBT was standard CBT delivered by therapists over the Internet and consisted of CBT components, such as behavioral activation and cognitive restructuring, and partly by providing preventive strategies and skills (e.g. mindfulness exercises)	ICBT significantly reduced the proportion of relapse (*p* = 0.006). Low effect sizes were noted on the MADRS and BDI II (0.29–0.33)	
Titov, Andrews, Davies, et al. (2010)	117 patients with DSM IV depression were randomized to receive ICBT which was either clinician assisted (CA; *n* = 41) or technician assisted (TA; *n* = 37) or wait-listed (WL; *n* = 39). Assessed with The PHQ-9, Beck Depression Inventory (BDI-II), theKessler 10 (K-10), the SDS, and the Credibility/Expectancy Questionnaire (CEQ) at baseline, one week post-treatment and at four months post-treatment (follow-up)	Treatment group participants received access to the Sadness program, an ICBT program consisting of six online lessons, printable summary and homework assignments, automatic e-mails, and additional resource documents	No significant differences noted in between-group effect sizes (TA vs. CA) on any measure (Cohen's *d* = 0.07) at completion of treatment. However, when compared to control, both groups had robust effect sizes (0.9–1.3)	
		The TA group received assistance from a technician employed as an administrator while the CA group did so from a psychiatrist	At follow-up however, the TA group had more modest increase in effect size compared to CA group (0.40–0.45). Follow-up at 4 months	
Wagner et al. (2014)	62 participants suffering from depression were randomly assigned to the therapist-supported IBI group (*n* = 32) and to the face-to-face intervention (*n* = 30). Primary outcome measure was the Beck Depression Inventory-II (BDI-II)	Online and face-to-face intervention groups received a brief (8 weeks) cognitive-behavioral therapy (CBT) program for depression and involved the following CBT modules: (1) introduction, (2) behavioral analysis, (3) planning of activities, (4) daily structure, (5) life review, (6) cognitive restructuring, (7) social competence, and (8) relapse prevention	The intention-to-treat analysis yielded no signiﬁcant between-group difference (online vs. face-to-face group) for any of the pre- to post-treatment measurements. At post-treatment both treatment conditions revealed signiﬁcant symptom changes compared to before the intervention. Within-group effect sizes for depression in the online group (*d* = 1.27) and the face-to-face group (*d* = 1.37). Pre-treatment to 3-month follow-up within-group effect sizes on the BDI were more favorable for the online-group (*d* = 2.00), compared to the face-to-face group (*d* = 1.04)	Small sample size
Andersson, Hesser, Veilord, et al., (2013)	Participants with mild to moderate depression were recruited from the general population and randomized to either guided ICBT (*n* = 33) or to live group treatment (*n* = 36). The MADRS-S was used as the main outcome measure of depression at the different assessment points. In addition, the BDI was used as a secondary depression measure	The self-help treatment consisted of seven text modules (introduction to CBT, depression from a CBT-perspective with a behavioral focus, behavioral activation, cognitive restructuring, sleep management, defining goals/values, and relapse prevention	The within-group standardized effect sizes for ICBT were significant, with Cohen's *d* = 1.46 (CI 95% = 0.89 to 2.02) at post-treatment and *d* = 0.99 (CI 95% = 0.51 to 1.47) at 3-year follow-up. The between-group SMD (Cohen's d) at post-treatment was *d* = 0.58 (CI 95% = 0.09 to 1.05), and at the 3-year follow-up *d* = 0.55 (CI 95% = 0.06 to 1.02), with both being in favor of ICBT	
Johansson et al. (2013)	One hundred participants with diagnoses of mood and anxiety disorders participated in a randomized (1 : 1 ratio) controlled trial of an active group vs. a control condition. Outcome measures were the 9-item Patient Health Questionnaire Depression Scale (PHQ-9) and the 7-item GAD scale (GAD-7). All measures were administered weekly during the treatment period and at a 7-month follow-up	The treatment group received a 10-week, psychodynamic, guided self-help treatment based on Affect-phobia therapy (APT) that was delivered through the Internet. The treatment consisted of eight text-based treatment modules and included therapist contact (9.5 minute per client and week, on average) in a secure online environment	A large between-group effect size of Cohen's *d* = 0.77 (95% CI: 0.37–1.18) was found on the PHQ-9. Follow-up at 7 months	Substantial within-group effects in the control group make the results harder to interpret
Johansson et al. (2012)	Ninety-two participants who were diagnosed with Major Depressive Disorder according to the Mini-International Neuropsychiatric Interview were randomized to treatment or an active control. The primary outcome measure was the Beck Depression Inventory-II (BDI-II) that was administered pre-treatment, on a weekly basis during the entire treatment phase, at post-treatment and also 10 months after the treatment had ended	The psychodynamic treatment was given as guided self-help, with minimal text-based guidance provided on a weekly basis. In all, there were nine treatment modules, totaling 167 pages of text. The treatment modules were largely derived from the self-help book Make the leap that is based on psychodynamic principles. The treatment was called SUBGAP, which stands for (1) Seeing unconscious patterns that contribute to emotional difficulties, (2) Understanding these patterns, (3) Breaking such unhelpful patterns, and (4) Guarding Against Patterns and/or relapses	Between-group effect size on the BDI was large post-treatment (Cohen's *d* = 1.11; 95%CI = 0.67–1.56). Follow-up at 10 months	
Van Voorhees et al. (2009)	84 adolescents (14–21 years) with sub-threshold depression assessed using Center for Epidemiologic Studies Depression Scale (CES–D) scale were randomized to receive either Motivational Interviewing plus IBI (MI) or brief advice plus IBI (BA). Diagnosable psychiatric illness was excluded	The intervention comprises 14 modules based on behavioral activation, CBT, interpersonal psychotherapy, and a community resiliency concept model. The brief advice was a physician-delivered 2–3 minutes advice while the MI was a longer 10–15 minute interview to engage the client	Primary depressive disorder and symptom outcomes at 6 and 12 weeks were similar between-groups. Moderate effect sizes were noted on CES-D (MI-0.56; BA-0.82)	
Warmerdam et al. (2010)	263 participants with clinically significant depressive symptoms (16 or more on CES-D) were randomized to Internet-based CBT (*n* = 88), Internet-based problem-solving therapy (*n* = 88), and a waiting list (*n* = 87). End points were evaluated at the 12-week follow-up. Assessed with by the Center for Epidemiological Studies Depression (CESD) scale – Dutch version and EQ-5D of the EuroQol group. Costs were obtained with the Trimbos and Institute of Medical Technology Assessment Cost Questionnaire for Psychiatry	The Internet-based problem-solving therapy had six steps: describing the problem, brainstorming, choosing the best solution, making a plan for carrying out the solution, actually carrying out the solution, and evaluation, over a period of 5 weeks. CBT in this study included psycho-education and focused on skills such as relaxation, cognitive restructuring (including coping with worrying thoughts), social skills training, and behavioral activation, specifically increasing the number of pleasant activities, over a period of 8 weeks. Both groups were therapist supported	Costs were similar across all three groups. While it costs 2800 euros for CBT per person, it was 2700 Euros for PST and 2560 Euros for the wait-listed group	High attrition rates
				Costs estimated for only treatment period (12 weeks)
				Self-report which may have recall bias
Warmerdam et al. (2008)	263 participants with depressive symptoms (≥16 on the Center for Epidemiological Studies Depression scale) were randomized to the three conditions (CBT: *n* = 88; PST: *n* = 88; WL: *n* = 87). Assessed with Center for Epidemiological Studies Depression scale (CES-D, Hospital Anxiety and Depression Scale (HADS), EuroQol Questionnaire (EQ5D)	PST subjects described their problems and wrote them down. They then divided these problems into three categories: (a) unimportant problems (problems unrelated to the things that matter to them), (b) solvable problems, and (c) problems which cannot be solved (e.g. the loss of a loved one). Then problem-solving strategies or coping measures were suggested by six-step procedure: describing the problem, brain-storming, choosing the best solution, making a plan for carrying out the solution, actually carrying out the solution, and evaluation. The course took 5 weeks and consisted of one lesson a week.	Between-group effect sizes were modest (0.54)	High attrition rates (30%)
		CBT is based on Coping with Depression course		
Farrer et al. (2011)	105 callers to a national helpline service with moderate to high psychological distress (22 or above on the 10-item Kessler Psychological Distress Scale (K10)) were recruited and randomized to receive either Internet CBT plus weekly telephone follow-up; Internet CBT only; weekly telephone follow-up only; or treatment as usual. Assessed using the Center for Epidemiologic Studies Depression Scale at pre, post, and 6 months follow-up	The web-only intervention delivered online psycho-education (in Week 1, provided by BluePages: bluepages.anu.edu.au) combined with CBT (in weeks 2–6, provided by MoodGYM: moodgym.anu.edu.au). The telephone assistance was a weekly 10-minute telephone call from a telephone counselor, with the call addressing any issues associated with the participants' use of the online programs	Between-group effect sizes were the highest for the Internet CBT plus weekly telephone follow-up (1.04) followed by ICBT only (0.76), and Telephone calls only (0.38). The effect sizes kept improving at 6 months to become more robust. Follow-up at 6 months	Only single assessment tool.
Spek, Nyklicek et al. (2007)	In a sample of 301 participants aged above 50 years with sub-threshold depression diagnosed as a score of above 12 on the Edinburgh Depression Scale (EDS) were randomized into three groups: Internet-based CBT, group CBT and Wait-listed control group. Assessed using the BDI as outcome measure at the end of 10 weeks	The group cognitive behavior therapy protocol was the Coping With Depression (CWD) course consisting of 10 weekly group sessions on psycho-education, cognitive restructuring, behavior change, and relapse prevention. The Internet-based CBT comprised eight modules with text, exercises, videos and figures based on the CWD protocol without any therapist help	The group CBT condition had a large improvement effect size of 0.65, while an even larger improvement effect size of 1.00 was found within the Internet-based treatment condition	High drop-out rates (52%) among Internet CBT group. Also included people with high education levels, which may have influenced results
van der Zanden et al. (2012)	244 young people with depressive symptoms were randomly assigned to the online MYM course or to a waiting-list control condition. The primary outcome measure was treatment outcome after 3 months on the Center for Epidemiologic Studies Depression Scale	The online MYM group course is a structured form of CBT for depression. At the core of MYM is the cognitive restructuring of thinking patterns. Course participants are encouraged to detect their own unproductive, unrealistic thoughts, and they are then taught to transform these into realistic, helpful thoughts. Performance of pleasant daily activities is also encouraged, and a mood measure is filled in daily to help understand the connection between pleasant activities and the mood level	Between-group effect sizes were moderately large at the end of 12 weeks (*d* = 0.94; *p* < 0.001), which persisted at 24 weeks (*d* = 1.97; *p* < 0.001)	
Carlbring et al. (2013)	A total of 80 individuals from the general public were randomized to one of two conditions: treatment or control. Both groups completed a weekly mood rating by answering the nine items on the MADRS-S. The primary outcome measure was the beck Depression Inventory II (BDI-II)	The treatment material used in this study was a commercially available program called “Depressionshjälpen”. The program has a focus on behavioral activation with influences from Acceptance and Commitment therapy (ACT). The nine modules consisted of Psycho-education about depression, link between activity and well-being, Understanding different activities and the role of reinforcement, Make a difference in your life, Thoughts and emotions, Repetition and continued practice, and relapse prevention	On the main outcome measure (BDI-II) the between-group effect size was *d* = 0.98 (95%CI = 0.51–1.44). Treatment gains were maintained at the 3-month follow-up	Comparison with control sample not carried out at 3 months
Donker et al. (2013)	This automated, 3-arm, fully self-guided, online non-inferiority trial compared two new Internet-delivered treatments (IPT and CBT) to an active control treatment delivered online (MoodGYM) for depressed individuals. 1843 participants were eventually randomized into 1 of the three groups. The 20-item self-report CES-D was used to assess depressive symptoms and was the primary outcome measure	The Internet-delivered CBT intervention comprised one component of the depression stream of e-couch and consisted of three major modules: identifying negative thoughts, tackling negative thoughts, and undertaking behavioral activation. The program contained 18 exercises and assessments in total	Intention to treat analyses revealed low effect size differences between all three arms (Cohen's *d* = 0.09 for IPT vs. MoodGym and 0.01 for CBT vs. Moodgym)	
		The Internet-delivered form of IPT comprised one component of the depression stream of e-couch and consisted of four modules (grief, role disputes, role transition, and interpersonal deficits) and a personal workbook (containing 13 exercises and assessments)		
		The online CBT package comprised a 4-module version of MoodGYM delivered over 4 weeks

**Table 2.** RCTs in depression without therapist guidance.

Vernmark et al. (2010)	As detailed in Table 1			
Spek, Nyklicek, Smits, et al. (2007)	As detailed in Table 1			
De Graaf et al. (2009)	Three hundred and three people in the Netherlands with depression (BDI–II) score > 16) were randomly allocated to one of three groups: Colour Your Life; treatment as usual (TAU) by a general practitioner; or Colour Your Life and TAU combined. Assessments included BDI II, Symptom Checklist 90 (SCL–90), Work and Social Adjustment Scale, 36-item short-form Health Survey (SF–36) at baseline and at 6-month follow-up	Colour Your Life is an online, multimedia, interactive computer program for depression consisting of eight 30-min sessions and a ninth booster session, with homework assignments	No significant differences on the primary outcome measure as well as most secondary outcomes (all *p *> 0.29) except for the Work and Social Adjustment Scale. Between-group effect sizes were trivial (0.02–0.06). Treatment adherence was also low in all interventions. Follow-up at 6 months	More severely depressed patients and low adherence levels are major limitations
Andersson et al. (2005)	117 participants with mild to moderate depression (<30 on MADRS-S) were randomized to receive either Internet-based CBT with minimal therapist contact or Wait-listed group. Assessments included 21-item Beck Depression Inventory (BDI), MADRS-S (9 items), the 21-item Beck Anxiety Inventory (BAI), and the Quality of Life Inventory (QoLI) pre, post, and at 6 months follow-up	Five modules: introduction; behavioral activation; cognitive restructuring; sleep and physical health; and relapse prevention, and future goals over 8–10 weeks	Effect sizes (Cohen's *d* between-groups at post-treatment) were 0.94 for the BDI, 0.79 for the MADRS–S, and 0.47 for the BAI at post-treatment. Follow-up at 6 months	No comparator group at 6 months follow-up
Bolier et al. (2013)	A 2-armed RCT that compared the effects of access to Psyfit for 2 months (*n* = 143) to a waiting-list control condition (*n* = 141) was conducted among mild to moderately depressed adults in the general population. Secondary outcomes were depressive symptoms measured by Center for Epidemiological Studies Depression Scale (CES-D). Online measurements were taken at baseline, 2 months, and 6 months after baseline	Psyfit is an online self-help intervention, without support from a therapist. The intervention is based on positive psychological principles and addresses strengths and personal competencies rather than mental problems and deficiencies. It incorporates evidence-based exercises based on positive psychology and elements stemming from mindfulness, CBT, and problem-solving therapy	Depressive symptoms showed modest changes in within-group effect size at 2 months (Cohen's *d* = 0.36, *p *= 0.02) and at six months (Cohen's *d* = 0.35, *p* = 0.02). Follow-up at 2 and 6 months	No blinding of controls.
		There are six modules in Psyfit, each containing a 4-lesson program: (1) personal mission statement and setting your goals, (2) positive emotions, (3) positive relations, (4) mindfulness, (5) optimistic thinking, and (6) mastering your life. Each week, the lesson consisted of psycho-education and a practical exercise		
Clarke et al. (2009)	*299 participants with depression were randomized to receive either* access to the experimental Internet site (the intervention condition; *n* = 144 and the other 155 were not granted access (the usual care control condition) and assessed using the Center for Epidemiological Studies Depression Scale (CES-D)Scale at enrollment and at 4-, 8-, 16-, and 32-weeks after enrollment	The Internet intervention was a self-paced, skills training program focusing on the acquisition and use of cognitive restructuring techniques adapted from group CBT psychotherapy. No therapist was available for the entire duration of the study	No differences between the control and experimental group on self-reported depression (CES-D) over the study period were noted	Attrition rates were nearly 50% possibly due to the presence of more severely depressed patients
O'Kearney et al., (2006)	78 boys age 15 and 16 years were allocated to either undertake MoodGYM or to standard personal development activities. Assessments included Center for Epidemiological	Participants in the intervention group received MoodGYM, a self-paced interactive Internet program (www.MoodGYM.anu.edu.au) that aims to help people identify problems with depression, and gives information, demonstrations, questionnaires (e.g. about depression and anxiety levels) and practice exercises (e.g. relaxation, problem-solving, cognitive restructuring, assertiveness, self-esteem training and coping with relationships)	Low effect sizes (0.1–0.2) were noted on all measures	High attrition rates
	Studies Depression Scale (CES-D), Revised Children's			Small sample size
	Attributional Style Questionnaire (CASQ-R), Rosenberg Self-Esteem Scale (RSES), Depression Stigma Scale			Non-randomized study
Lintvedt et al. (2011)	163 students (mean age 28.2 years) with elevated psychological distress (20 or above on Kessler Psychological Distress Scale (K10) were recruited to the trial and randomized to an Internet intervention condition or the waiting list control group. Assessed with K10, Center for Epidemiologic Studies Depression Scale (CES-D), Automatic Thoughts Questionnaire (ATQ) and TDL (treatment depression literacy)	Intervention group received access to the Bluepages and Moodgym CBT program	Between-group effect sizes on ITT analyses were modest for CES D (0.57) and ATQ (0.50). The effect size improved to 0.74 when only completers were analyzed	47% drop out rates!!
				Unguided interventions may have led to drop outs and hence lower effect sizes.
				No longer term follow-ups
(Spek et al. (2008)	301 participants with sub-threshold depression (Edinburgh Depression Scale (EDS) score of 12 or more but no DSM IV diagnosis) were randomized into Internet-based treatment, group CBT (Coping with Depression Course), or a waiting-list control condition. Assessed with BDI-21	Coping with Depression Course consists of 10 weekly group sessions on psychoeducation, cognitive restructuring, behavior change, and relapse prevention. The Internet-based CBT is an intervention of eight modules, is self-participatory and has no therapist support	Modest effect size noted with Internet-based treatment (0.5) but no difference between-group CBT and wait-list	37% drop out rates
Meyer et al. (2009)	216 adults with self-reported depressive symptoms randomized by 80 : 20 design into the Deprexis program or wait-listed. Assessed with BDI, Work and Social Adjustment Scale	The Web-based intervention (Deprexis) consists of 10 content modules representing different psychotherapeutic approaches, plus one introductory and one summary module. The modules’ theoretical rationale and content draws from theories like (1) Behavioral Activation, (2) Cognitive Modification, (3) Mindfulness and Acceptance, (4) Interpersonal Skills, (5) Relaxation, Physical Exercise and Lifestyle Modification, (6) Problem Solving, (7) Childhood Experiences and Early Schemas, (8) Positive Psychology Interventions, (9) Dreamwork and Emotion-Focused Interventions, and (10) Psycho-education	Between-group effect size was modest (0.36). It improved however to a more robust 0.74 at 6 months follow-up	No screening procedure detailed
				High attrition rates
Christensen et al. (2004)	435 participants with 22 or above on the Kessler psychological distress scale were randomized into one of three groups: Blue Pages, MoodGym (ICBT) or Control group. Assessed with Center for Epidemiologic Studies- Depression Scale	Bluepages was an information-only website giving literacy on depression, MoodGym was an online CBT program and the Control group was wait-listed	Both Bluepages and Moodgym had modest effect sizes (0.4). However, patients with more severe depression showed higher effect sizes (0.8–0.9)	
Ünlü Ince et al. (2013)	96 Turkish adults with depressive symptoms were randomized to the experimental group (*n* = 49) or to a wait-list control group (*n* = 47) and administered the CES-D for evaluating depression severity. The treatment group received the self-guided, problem-solving intervention – Turkish version (AOC-TR) which was administered in five sessions over five weeks. The control condition was a waiting list comparator. Participants were assessed online at baseline, post-test (6 weeks after baseline), and 4 months after baseline. Post-test results were analyzed on the intention-to-treat sample	The AOC-TR consists of five sessions over 5 weeks. During the intervention, participants indicate what they think is important in their lives, they make a list of their problems and worries, and they categorize their problems into three groups: (1) unimportant problems, which are not related to what they think is important in their lives, (2) important and solvable problems, which are approached by a systematic problem-solving approach consisting of six steps, and (3) important but unsolvable problems, such as having lost someone through death or having a chronic general medical disease and making a plan for how to live with it. The core of the intervention is the 6-step problem-solving procedure, which teaches to use this technique during the course for several of their important and solvable problems. The idea is that by mastering this technique people will regain mastery of their problems and ultimately their lives	Within-group effect size was non-significant for the depression group (Cohen's D-0.37 (CI-0.03–0.78). Follow-up at 4 months	Small sample size
				Using Facebook as major recruitment strategy

**Table 3.** Non-RCT non-CBT interventions in depression.

Lipman et al., (2011)	15 lone mothers were recruited and involved in a pilot study to improve coping and mood using Web-based video conference group cognitive therapy. Assessments included Center for epidemiological Studies Depression Scale [CES-D], Rosenberg Self-Esteem Scale, Social Provisions Scale and Parenting Stress Index-Short Form at pre and post was done. In addition, a focus group discussion of seven women was also carried out	Group video conferencing involving cognitive behavioral techniques and structured group counseling that included both child and maternal themes	Nonsignificant improvements were observed in all measures. However, qualitative analysis of the technique was overwhelmingly positive	Small sample size
				The provision of a free computer and free Internet for a year may have influenced participants to be positive about the intervention
Van Voorhees et al., (2005)	14 late adolescents (ages 18–24) with at least one risk factor for developing depression (personal or family history of a depressive episode) were included. Assessments included Center for Epidemiologic Studies of Depression Scale (CES-D), Automatic Thoughts Questionnaire Revised (ATQ), and Social Support Questionnaire – Short Form, (SSQ-6)	The intervention included an initial motivational interview in primary care, 11 Web-based modules based on CBT and IPT and a follow-up motivational interview in primary care to enhance behavior change	Moderate effect sizes in depressive symptoms (0.43) and low effect sizes on the other two measures were noted (0.17–0.27)	Small sample size
				No active comparator
Andersson, Hesser, Hummerdal, et al. (2013)	3.5-year post-treatment follow-up of two versions of ICBT (Internet-delivered self-help vs. e-mail therapy) for mild to moderate depression on 51 participants. Assessed with the 21-item Beck Depression Inventory (BDI), 21-item Beck Anxiety Inventory (BAI), and the Quality of Life Inventory (QOLI)		The pre-treatment to 3.5-year follow-up within-group effect size was *d* = 1.7 for guided self-help and *d* = 1.5 for e-mail therapy on the BDI showing a sustained response. Mild improvements noted on QOLI	Dropout rate was 42%
				Not controlled for additional treatment
Mohr et al. (2010)	*19 patients with depression (*10 and above on the PHQ-8) received the “moodManager”, which was based on cognitive behavioral principles and consisted of six learning modules and four tools and was monitored by a coach. Assessed with the Hamilton Rating Scale for Depression (HRSD), Personal Health Questionnaire (PHQ-9), Perceived Barriers to Psychological Treatment (PBPT), GAD scale (GAD-7), Telephone Interview for Cognitive Status (TICS) and the 10 self-report items from the Positive Affect Scale of the Positive and Negative Affect Scale (PANAS)	Learning modules (and associated tools) included the following: (1) “Getting Started”, which was an introduction to the basic principles of CBT; (2) “Monitoring Activities”, which described the relationship between activities and mood and introduced the “Activity Diary” tool, which allowed participants to track and rate daily activities; (3) “Scheduling Positive Activities”, which taught participants to use the “Activity Scheduler”, a tool used to plan and schedule positive activities; (4) “Identifying Thoughts”, which described the effects of thoughts on mood and taught participants to use the “Thought Diary” tool to monitor automatic thoughts; (5) “Challenging Thoughts”, which expanded the Thought Diary tool by teaching participants to develop alternative thoughts; (6) “Maintaining Gains”, which summarized the skills learned and encouraged participants to continue using the tools for relapse prevention	Within-group effect size was high (1.34 for HRSD, 1.96 for PHQ 9, 1.70 for GAD-7) at the end point.	No comparator group
				Small sample

**Table 4.** RCTs in anxiety disorders with therapist guidance.

Berger et al. (2011)	Recruitment done online and of 275 volunteers with Social Phobia, 81 included who met cut-offs on the Social Phobia Scale (SPS) and Social Interaction Anxiety Scale (SIAS). Randomized to three different groups: Pure Internet-based self-help program; self-help plus contact with therapist; self-help and support on demand. Assessed with the SPS, SIAS and the Liebowitz Social Anxiety Scale (LSAS-SR), BDI-II, Brief Symptom Inventory (BSI) and the Inventory of Interpersonal Problems (IIP) at pre, post, and 6 months follow-up	Online lessons comprising five largely text-based lessons, several exercises and diaries (e.g. negative thoughts diary), and the possibility to participate in an online discussion forum. Lesson 1: Motivational interview; Lesson 2: psycho-education and CBT model; Lesson 3: Cognitive restructuring; Lesson 4: Cognitive exercises; Lesson 5: Behavioral experiments in vivo. All lessons are done over 10weeks	Significant improvements in all groups from pre- to post-treatment and from pre-treatment to follow-up (*p* < 0.001 on every measure), but no group showed additional improvement between post-treatment and follow-up (all *p*’s > 0.05). Moderate effect size difference (0.3) between Guided and self-help on LSAS-SR and IIP. Moderate effect size of 0.33 between Step up vs. self-help on BDI. No other effect sizes significant enough. Follow-up at 6 months	Small sample size.
				No control group without any intervention
Hedman et al. (2011b)	Cost effective analysis of Internet-based CBT compared to cognitive behavior group therapy CBGT for Social phobia from a societal perspective within the context of a RCT of 126 participants	Costs were calculated using the Trimbos and Institute of Medical Technology Assessment Cost Questionnaire for Psychiatry (TIC-P). The TIC-P covers monthly direct medical costs, i.e. health-care consumption (e.g. GP visits) as well as nondirect medical costs, i.e. costs of other health-related services not directly associated with healthcare (e.g. time spent in self-help groups)	ICBT was cheaper by 2000 USD to CBGT in both post-treatment and follow-up costs	Absence of any control group
				Self-assessment of costs reduce objectivity
Carlbring et al. (2011)	54 participants with DSM IV diagnosis of any specific anxiety disorder, or anxiety disorder NOS, having only moderate depression were assessed using Clinical Outcomes in Routine Evaluation e-Outcome Measure (CORE-OM), the self-rated version of the Montgomery-Åsberg Depression Rating Scale (MADRS-S), Beck Anxiety Inventory (BAI), Quality in life inventory (QOLI) and randomized into two groups: Treatment and Control group. All assessments were repeated post-test and at 1 and 2 years follow-up	A combination of CBT modules for panic disorder, social phobia and GAD, and depression was prescribed (6–10 modules over 10 weeks) and individual feedback given over e-mail	Effect sizes of 0.67–1 were noted for all measures post-test between Treatment and Control group. These measures improved slightly at 1 and 2 years follow-up	Mixed diagnosis group and mixed interventions provided
Titov et al. (2011)	Australians meeting DSM IV diagnosis of either depression/GAD/SP or Panic disorder were randomized into Treatment (*n* = 32) and Waiting groups (*n* = 35)	Administered MINI to confirm diagnosis. Intervention consisted of eight online lessons (Lesson 1: Psycho-education, Impact of illness, Normalization; Lesson 2: CBT principles, challenging thoughts, Shifting attention; Lesson 3: De-arousal, Scheduling lifestyle, managing panic; Lesson 4: Behavioral activation; Lesson 5: Graded exposure ; Lesson 6: Addressing cognitive beliefs; Lesson 7: Problem solving; Lesson 8: Relapse prevention, homework assignments for each lesson, an online discussion forum for each lesson, moderated by the therapist; regular automatic reminder and notification e-mails; and instant messaging to allow secure e-mail-type messages with a clinician. Assessed with PHQ 9, Penn State Worry Questionnaire, Social Phobia-12 (SP-12), Panic Disorder Severity Scale-Self Rating (PDSS-SR), GAD-7-item scale, (GAD-7), Kessler-10 item (K-10), SDS and NEO-Five Factor Inventory-Neuroticism Subscale (NEO-FFI-N), at three time points: pre-, 1 week post- and 3 months post-treatment	Post-treatment DASS-21, PHQ-9, PSWQ, and PDSS-SR scores, controlling for pre-treatment scores, revealed that the Treatment group had significantly lower post- treatment scores than the Control group, (F1.71 = 13.22, *p* < 0.001), (F1.71 = 10.32, *p* < 0.002), (F1.71 = 11.40, *p* < 0.001), (F1.71 = 5.47, *p* < 0.02), respectively. Also, Treatment group had significantly lower post-treatment scores than the Control group on the GAD-7 (F1.71 = 9.02, *p* < 0.004), K-10 (F1.71 = 8.74, *p* < 0.004), NEO-FFI-N (*F*1.71 = 7.63, *p* < 0.007) and SDS (*F*1.71 = 4.10, *p* < 0.05). No differences in any measure between post-treatment and follow-up. Moderate between-group effect sizes (0.5) noted on DASS 21, PHQ 9 and PSWQ	Small sample size limits the findings. In addition, the control group was not assessed as many times as the Treatment group, which could have controlled for other confounders
Hedman, Andersson, Andersson et al. (2011)	81 people with DSM IV hypochondriasis and have only mild-moderate depression were randomized to receive either ICBT or Wait-listed. Health Anxiety Inventory (HAI), Illness Attitude Scale (IAS), Whiteley Index, Beck Anxiety Inventory (BAI), Anxiety Sensitivity Index (ASI), MADRS–S16 and the Quality of Life Inventory (QOLI) were used as measures pre, post and at 6 months follow-up	12 Internet modules comprising role of avoidance and safety behaviors, internal focus, and interpretations of bodily sensations and mindfulness training, given over 12 weeks with online therapist support available	Significant effect between the Internet-based CBT group and the control group on HAI (*F* = 95.90, df. = 1,78, *p* < 0.001), IAS (*F* = 92.70, df. = 1,78, *p* < 0.001) and Whiteley Index (*F* = 59.15, df. = 1,78, *p* < 0.001) at post-treatment were observed. Effect sizes ranged from 1.05 to 1.62 at post-treatment. Follow-up at 6 months	No comparator group at 6 months follow-up
Andrews et al. (2011)	25 participants with Social phobia were randomized to receive either ICBT or face-to-face CBT. Assessments included Social Interaction Anxiety Scale (SIAS), the Social Phobia Scale (SPS) and the World Health Organization Disability Assessment Schedule (WHODAS2), pre and post	The ICBT program was the Shyness program and comprised six online lessons (lesson 1 and 2: Psycho-education; Lesson 3: Exposure hierarchy; Lesson 4 and 5: Graded exposure; Lesson 6: Relapse prevention), a summary/homework assignment for each lesson; comments by participants on a forum moderated by the clinician (M.D.); access to supplementary materials; automatic e-mails, and fortnightly short message service (SMS), to be completed over 8 weeks. Face-to-face CBT was a group CBT weekly for seven weeks for 4 h under the guidance of the same clinician (M.D.) following the same principles	No differences between the ICBT and face-to-face CBT group on the SIAS or SPS and the WHODAS2	Small sample size
Paxling et al. (2011)	89 participants from the general community with DSM IV GAD were randomized into receiving either Internet-delivered CBT or a wait-listed group. Assessed with Penn State Worry Questionnaire (PSWQ), the primary outcome measure, GAD questionnaire-IV (GAD-Q-IV), Montgomery–Asberg Depression Rating Scale – Self-Rated (MADRS-S), Alcohol-Use Disorders Identification Test (AUDIT) and the CGI at pre, post, 1 year and 3 years follow-up	The CBT program consisted of therapist-guided Internet-delivered eight text-based treatment modules delivered on a weekly basis for 8 weeks. Briefly, they were (1) psycho-education (2) Step 1 of applied relaxation (3) Step 2 of applied relaxation and worry time (4) Step 3 of applied relaxation and cognitive restructuring (5) Step 4 of applied relaxation, more on cognitive distancing and problem solving (6) Step 5 of applied relaxation and worry exposure (7) Step 6 of applied relaxation, interpersonal problem solving, and sleep management and (8) relapse prevention	Between-group effect sizes at end point and follow-up were robust for PSWQ (1.11–1.66), GADQ (1.07–1.65), BDI (0.86–1.11), BAI (0.85–1.32) and MADRS (0.98–1.42). Follow-up at 1 and 3 years	Not all completed the entire module
Titov et al. (2008)	98 individuals with social phobia were randomized into three groups: therapist-assisted (CaCCBT), self-guided (CCBT), or to a wait-list control group. Assessments included Social Interaction Anxiety Scale (SIAS), Social Phobia Scale (SPS), the Patient Health Questionnaire Nine-Item (PHQ-9), the Kessler 10(K-10) and the SDS (Wagner et al., 2014)	CaCCBT group received the Shyness treatment program consisting of six online lessons; homework assignments; participation in an online discussion forum; and regular e-mail contact with a therapist.	Large effect sizes were observed between the CaCCBT and control groups on the SIAS (0.99) and SPS (1.08). Moderate effect sizes were found between the CaCCBT and CCBT groups on the SIAS (0.64) and SPS (0.67), and small effect sizes were observed between the CCBT and control groups on the SIAS (0.34) and SPS (0.41)	
		Lessons 1 and 2: Psycho-education; Lesson 3:		
		Exposure hierarchy and graded exposure; Lessons 4 and 5: reinforce principles of graded exposure and principles of cognitive restructuring; Lesson 6: Relapse prevention. CCBT group received the Shyness treatment program as described above, but without regular e-mails or forum responses from the therapist		
Titov, Andrews, Choi, Schwencke, and Johnston, (2009)	Data from three RCTs using the Shyness program to treat social phobia were reanalyzed. The 211 subjects, all of whom met DSM-IV criteria for social phobia, were divided into four groups: (i) social phobia only (SP); (ii) social phobia with elevated symptoms of depression (SP + Dep); (iii) social phobia with elevated symptoms of generalized anxiety (SP + GAD); and (iv) social phobia with elevated symptoms of both generalized anxiety and depression (SP + Dep + GAD)	The improvement in social phobia (Social Phobia Scale (SPS) and Social Interaction Anxiety Scale (SIAS), depression (Patient Health Questionnaire nine-item (PHQ-9), and anxiety (GAD-7-Item Scale (GAD-7) following Internet-based CBT (Shyness program) for social phobia was measured	Within-group effect sizes were large for all groups on the SIAS (1.1–1.7) and SPS (1.0–1.2). Large effect size differences were also noted for the SP + Dep + GAD group on PHQ 9 and GAD-7. Other results were small or inconsistent	
Robinson et al., (2010)	138 patients with DSM IV GAD were randomized to receive ICBT which was either clinician assisted (CA; *n* = 46) or technician assisted (TA; *n* = 45) or wait-listed (WL; *n* = 47). Assessed with The PHQ-9, the GAD-7-Item Scale (GAD-7) Penn State Worry Questionnaire (PSWQ), the Kessler 10 (K-10), the SDS, and the Credibility/Expectancy Questionnaire (CEQ) at baseline, one week post-treatment and at three-month post-treatment (follow-up)	Treatment group participants received access to the Worry program, an iCBT program consisting of six online lessons, printable summary and homework assignments, automatic emails, and additional resource documents	Robust between-group effect size noted compared to control for both groups at post-treatment (1.02–1.25). No significant differences between TA and CA at this point (Cohen's *d* = 0.07–0.11). At follow-up, effect sizes improved, more for TA than for CA (0.2–0.3). Follow-up at 3 months	
		The TA group received assistance from a techinican employed as an administrator while the CA group did so from a psychiatrist		
Hedman et al. (2011b)	126 individuals with DSM IV Social phobia were randomized to receive either Internet-based CBT (ICBT; *n* = 64) or Cognitive behavioral group therapy (CBGT; (*n* = 62), both with therapist guidance. Assessed with the Liebowitz Social Anxiety Scale (LSAS), Beck Anxiety Inventory (BAI), Anxiety Sensitivity Index (ASI), Montgomery Asberg Depression Rating Scale-self report (MADRS-S) and Quality of Life Inventory (QOLI).	ICBT comprises 15 text modules, each covering a specific theme (e.g. exposure or cognitive restructuring) completed with a homework component. The modules provided the participants with the same knowledge and tools as conventional individual CBT for SAD over a period of 15 weeks.	Modest between-group effects sizes were noted favoring ICBT (0.24–0.41), which was maintained at follow-up. Within-group effect size was large, both at post-treatment (0.7–1.4) and at follow-up (0.9–1.6).	No control group.
		CBGT comprised an initial individual session followed by 14 group sessions over 15 weeks.		
Johnston et al., (2011).	121 Individuals meeting DSM-IV criteria for a principal diagnosis of GAD, social phobia (SP) or panic disorder with or without agoraphobia (Pan/Ag) were randomized to receive ICBT which was either clinician assisted (CA; *n* = 42) or technician assisted (TA; *n* = 39) or wait-listed (WL; *n* = 40). Assessed with the GAD-7-Item Scale, (GAD-7), Depression Anxiety Stress Scales – 21 Item (DASS-21), Penn State Worry Questionnaire (PSWQ), Social Interaction Anxiety Scale and Social Phobia Scale – Short Form (SIAS-6/SPS-6), Panic Disorder Severity Scale – Self Rating (PDSS-SR), Patient Health Questionnaire – nine Item (PHQ-9) and SDS	Both treatment groups received access to the enhanced Anxiety Program comprising eight online lessons; a summary/homework assignment for each lesson; weekly telephone or e-mail/asynchronous messaging contact with the Clinician or Technician, and regular automated reminder and notification e-mails		
	Within-group effect sizes compared to control were modest to robust, more so for the TA group (1.06–1.3) than the CA group (0.7–0.9). Between-group effect size favored TA over CA group (0.3–0.5)	Non-blinded assessments		
		The TA group received assistance from a psychologist without specialist postgraduate training while the CA group did so from a psychologist with postgraduate training in Clinical Psychology and experience in ICBT		
Titov, Andrews, Johnston, Robinson, and Spence (2010)	Eighty-six individuals meeting diagnostic criteria for GAD, panic disorder, and/or social phobia (by MINI) were randomly assigned to a treatment group, or to a wait-list control group. Assessments included GAD-7-Item Scale, (GAD-7), Penn State Worry Questionnaire (PSWQ), Social Phobia Screening Questionnaire (SPSQ), the PHQ-9, the Kessler-10 item (K-10), SDS, Depression Anxiety Stress Scales (DASS-21) and the 12-item Neuroticism Subscale (NEO-FFI-N) of the NEO-Five Factor Inventory at pre, post, and 3 months follow-up	Treatment consisted of CBT-based online educational lessons (Lesson 1: Psycho-education, Lesson 2 : Controlling physical symptoms and the importance of lifestyle factors, Lesson 3: Basic principles of cognitive therapy, Lesson 4: Practicing graded exposure, Lesson 5: Education and guidelines about communication and assertiveness skills, Lesson 6: Relapse prevention) and homework assignments, weekly e-mail or telephone contact from a clinical psychologist, access to a moderated online discussion forum, and automated e-mails	Treatment group participants reported significantly reduced symptoms of anxiety as measured by the GAD-7 Item, Social Phobia Screening Questionnaire, and the Panic Disorder Severity Rating Scale, Self-Report Scale, but not on the Penn State Worry Questionnaire, with corresponding between-groups effect sizes (Cohen's *d*) at post-treatment of 0.78, 0.43, 0.43, and 0.20, respectively. Follow-up at 3 months	Mixed group of participants
Johansson et al. (2013)	One hundred participants with diagnoses of mood and anxiety disorders participated in a randomized (1:1 ratio) controlled trial of an active group vs. a control condition. Outcome measures were the 9-item Patient Health Questionnaire Depression Scale (PHQ-9) and the 7-item GAD scale (GAD-7). All measures were administered weekly during the treatment period and at a 7-month follow-up	The treatment group received a 10-week, psychodynamic, guided self-help treatment based on Affect-phobia therapy (APT), which was delivered through the Internet. The treatment consisted of eight text-based treatment modules and included therapist contact (9.5 min per client and week, on average) in a secure online environment	A moderately large between-group effect size *d* = 0.48 (95% CI: 0.08–0.87) was found on the GAD-7	Substantial within-group effects in the control group make the results harder to interpret
Carlbring et al. (2009)	30 months after completion of a previous RCT comparing ICBT and telephone support, 57 participants were contacted and assessed using the Liebowitz Social Anxiety Scale self-report version (LSAS-SR), the Social Phobia Scale (SPS), the Social Interaction Anxiety Scale (SIAS), Social Phobia Screening Questionnaire (SPSQ, Beck Anxiety Inventory (BAI), the self-rating version of the Montgomery Asberg Depression Rating Scale (MADRS-S) and the Quality of Life Inventory (QOLI)	Assessments made over Internet and using telephone	Pre-treatment to follow-up effect sizes ranged between *d* = 1.10 and 1.73 for the primary social anxiety measures, and between 0.47 and 0.82 for the secondary outcome measures

**Table 5.** (a) RCTs in anxiety disorders without therapist guidance, (b) Non-RCT/non-CBT interventions for anxiety disorders.

Titov, Andrews, Choi, Schwencke, and Johnston (2009)	As discussed in Table 4			
(a)				
Lorian et al. (2012)	44 individuals meeting diagnostic criteria for GAD were randomized to the ICBT treatment (*n* = 24) or control group (*n* = 20). Assessments included the GAD-7-Item Scale (GAD-7), Penn State Worry Questionnaire (PSWQ), Patient Health Questionnaire (PHQ9), Kessler Psychological Distress Scale (K10), Sheehan Disability Scale (SDS) and risk avoidance was assessed using Domain-Specific Risk Taking Scale (DOSPERT)	Treatment group participants received access to the Worry program, an ICBT program consisting of six online lessons, printable summary and homework assignments, automatic e-mails, and additional resource documents	Within the treatment group, medium to very large pre- to post-treatment effect sizes (Cohen's *d*) were indicated for measures of symptom severity and impairment (*d* = 0.62–1.67). Small to medium negative effect sizes were observed for social (*d* = −0.30) and recreational risk taking (*d* = −0.25)	Small sample sizes.
				Did not measure co-morbidities
Titov, Andrews, Choi, Schwencke, and Johnston (2009)	163 volunteers with social phobia (DSM IV) were randomized to receive either the computerized cognitive behavior therapy (CCBT) only or CCBT + telephone calls weekly. Assessments included Social Interaction Anxiety Scale (SIAS); the Social Phobia Scale (SPS); the PHQ-9; Kessler 10 (K-10) (Choi et al., 2012); and the SDS	The CCBT program involved six lessons which were: Lessons 1 and 2: Psycho-education; Lesson 3: Exposure hierarchy and graded exposure; Lessons 4 and 5: reinforce principles of graded exposure and principles of cognitive restructuring; Lesson 6: Relapse prevention. In addition, the other group also were telephoned each week by a research assistant, at a time specified by the participant, when they were commended and encouraged to persevere but no clinical advice was offered	Within-group effect size was moderate to robust, with the telephone group showing better improvement on the SIAS (1.4 vs. 0.98), SPS (0.89 vs. 0.73), SDS (0.83 vs. 0.79)	
Bolier et al. (2013)	A 2-armed RCT that compared the effects of access to Psyfit for 2 months (*n* = 143) to a waiting-list control condition (*n* = 141) was conducted among mild to moderately depressed adults in the general population. Secondary outcomes were anxiety symptoms measured by Hospital Anxiety and Depression Scale Anxiety subscale (HADS-A). Online measurements were taken at baseline, 2 months, and 6 months after baseline	Psyfit is an online self-help intervention, without support from a therapist. The intervention is based on positive psychological principles and addresses strengths and personal competencies rather than mental problems and deficiencies. It incorporates evidence-based exercises based on positive psychology and elements stemming from mindfulness, CBT, and problem-solving therapy	Anxiety symptoms showed modest changes in within-group effect size at 2 months (Cohen's *d* = 0.32, *P* = .001) and at six months (Cohen's *d* = 0.35, *P* = .001). Follow-up at 6 months	No blinding of controls.
		There are six modules in Psyfit, each containing a 4-lesson program: (1) personal mission statement and setting your goals, (2) positive emotions, (3) positive relations, (4) mindfulness, (5) optimistic thinking, and (6) mastering your life. Each week, the lesson consisted of psycho-education and a practical exercise		
(b)				
Hedman, Furmark et al. (2011)	5-year follow-up study of 80 persons with social phobia who had undergone Internet-based CBT. Assessments included Liebowitz Social Anxiety Scale-Self-Report (LSAS-SR), Social Interaction Anxiety Scale (SIAS), Social Phobia Scale (SPS), Social Phobia Screening Questionnaire (SPSQ), Montgomery-Åsberg Depression Rating Scale-Self-report (MADRS-S), Beck Anxiety Inventory (BAI), and Quality of Life Inventory (QOLI)	The CBT had included nine lessons: Introduction, Social anxiety model, cognitive restructuring, safety behavior experiments, exposure exercises, attention training, social skills, and relapse prevention. Also had feedback from a therapist	Within-group effect sizes of the LSAS-SR were large (1.30–1.40) at 5 years while all the others were moderate (0.7–1.0).	No comparator group at follow-up.
Rosmarin et al. (2010)	125 Jewish participants with subclinical anxiety, diagnosed as a score of 27 or higher on the Perceived Stress Scale and 54 or higher on the Penn State Worry Questionnaire. They were randomized into three groups: Spiritually integrated psychotherapeutic treatment (SIT), Progressive muscular relaxation (PMR), and Wait-listed Control. Assessments with PSS and PSWC along with Center for Epidemiological Studies Depression (CESD) Scale were done pre- and post-treatment	SIT consisted of cognitive (e.g. reading inspiring stories and excerpts from Jewish religious literature) and behavioral (e.g. spiritual exercises to increase gratitude and prayer) strategies from a spiritual perspective to improve anxiety	Scores on the Worry, stress, and depression scales significantly reduced, more for the SIT than the PMR compared to WLC [F(2. 91) = 12.15, *p* < 0.001, *R*^2^ = 0.21; *F*(2.92) = 5.82, *p* < 0.005, *R*^2^ = 0.11 and *F*(2.89) = 25.88, *p* < 0.001, *R*^2^ = 0.23), respectively	Specific measures with specific group only (those with high religiosity).
Dear et al. (2011)	Australians with MINI diagnosis of anxiety/depression (*n* = 32; depression = 18; GAD = 10; Panic = 2; Social phobia = 2) were placed in an open trial of ICBT derived from the Wellness program. Administered the DASS 21, PHQ 2, Penn State Worry Questionnaire (PSWQ), Social Interaction Anxiety Scale 6-item and Social Phobia Scale 6-item composite (SIAS6/SPS6), Panic Disorder Severity Scale –Self-Rating (PDSS-SR), GAD-7-Item Scale, (GAD-7), Kessler-10 Item (K-10), SDS and NEO-Five Factor Inventory e Neuroticism Subscale at pre, post, and 3 months follow-up	5 online lessons (Lesson 1: Psycho-education, Impact of illness, Normalization and assertive communication skills; Lesson 2: CBT principles, challenging thoughts, Shifting attention; Lesson 3: De-arousal, Scheduling lifestyle, managing panic; Lesson 4: Behavioral activation, Graded exposure, Problem solving; Lesson 5: Relapse prevention), a summary/homework assignment for each lesson; regular automatic reminder and instant messaging to allow secure e-mail-type messages with a clinician	Significant reductions on the DASS-21 (t31 = 5.89, *p* < 0.001), PHQ-9 (t31 = 6.54, *p* < 0.001), PSWQ (t31 = 5.99, *p* < 0.001), SIAS6/SPS6 (t31 = 2.92, *p* = 0.006), GAD-7 (t31 = 0.08, *p* < 0.001), *K*-10 (t31 = 0.36, *p* < 0.001), NEO-FFI-N (t31 = 5.42, *p* < 0.001) and SDS (t31 = 3.96, *p* < 0.001), but not on the PDSS-SR (t31 = 2.72, *p* < 0.11) between pre- and post-treatment. No differences between post-treatment and follow-up. Large within-group effect sizes (1.1) noted on DASS 21, PHQ 9 and GAD-7. Moderate effect sizes (0.6–0.8) noted on PSWQ, PDSS-SR, K-10 and NEO FFI-N	Completion rates of 81%
		Intention-to-treat analyses		41–73% of participants were classified as in remission at post-treatment and 50–59% were classified as recovered
Ünlü Ince et al. (2013)	96 Turkish adults with depressive symptoms were randomized to the experimental group (*n* = 49) or to a wait-list control group (*n* = 47) and administered the Hospital Anxiety and Depression Scale (HADS) for evaluating anxiety severity. The treatment group received the self-guided, problem-solving intervention – Turkish version (AOC-TR) which was administered in five sessions over five weeks. The control condition was a waiting list comparator. Participants were assessed online at baseline, post-test (6 weeks after baseline), and 4 months after baseline. Post-test results were analyzed on the intention-to-treat sample	The AOC-TR consists of five sessions over 5 weeks. During the intervention, participants indicate what they think is important in their lives, they make a list of their problems and worries, and they categorize their problems into three groups: (1) unimportant problems, which are not related to what they think is important in their lives, (2) important and solvable problems, which are approached by a systematic problem-solving approach consisting of six steps, and (3) important but unsolvable problems, such as having lost someone through death or having a chronic general medical disease and making a plan for how to live with it. The core of the intervention is the 6-step problem-solving procedure, which teaches to use this technique during the course for several of their important and solvable problems. The idea is that by mastering this technique people will regain mastery of their problems and ultimately their lives	Within-group effect size was non-significant for the anxiety group (Cohen's D-0.25 (CI -0.16–0.65). Follow-up at 4 months	Small sample size
				Using Facebook as major recruitment strategy

## Online interventions for depression

A total of 33 studies were selected, of which 26 involved the use of various CBT techniques including psycho-education, behavioral activation, cognitive restructuring, social skills training and relaxation, as well as problem solving and relapse prevention. Also, 29/33 studies were RCTs, with or without therapist guidance (which was defined as the presence of trained therapist involvement in the delivery of the intervention). The rest were either non-randomized studies that involved CBT or randomized studies that involved non-CBT techniques.

(a) RCTs (Tables 1 and 2): There were 29 RCTs involving online interventions for depressive disorders, this includes both with and without therapist guidance (Andersson et al., 2005; Andersson, Hesser, Veilord, et al., 2013; Bolier et al., 2013; Carlbring et al., 2013; Christensen et al., 2004; Clarke et al., 2009; Choi et al., 2012; De Graaf et al., 2009; Donker et al., 2013; Farrer, Christensen, Griffiths, & Mackinnon, 2011; Hollandare et al., 2011; Johansson et al., 2012, 2013; Kessler et al., 2009; Lintvedt et al., 2011; Meyer et al., 2009; O'Kearney et al., 2006; Perini, Titov, & Andrews, 2009; Ruwaard et al., 2009; Spek, Nyklicek et al., 2007; Spek et al., 2008; Titov, Andrews, Davies, et al., 2010; Ünlü Ince et al., 2013; Vernmark et al., 2010; Van Voorhees et al., 2009; Wagner, Horn, & Maercker, 2014; Warmerdam, Smit, van Straten, Riper, & Cuijpers, 2010; Warmerdam, van Straten, Twisk, Riper, & Cuijpers, 2008; van der Zanden, Kramer, Gerrits, & Cuijpers, 2012). These studies were studies from around the world; however Australia and Europe had major representation. Most studies involved the use of either the BDI or CES-D as assessment tools. Modules ranged from 6 to 12 weeks in length with follow-up periods ranging from 3 months to 5 years. Among the 29 RCTs, majority of the studies (19/29) were with the use of a therapist (Andersson, Hesser, Veilord, et al., 2013; Carlbring et al., 2013; Choi et al., 2012; Donker et al., 2013; Farrer et al., 2011; Hollandare et al., 2011; Johansson et al., 2012, 2013; Kessler et al., 2009; Perini et al., 2009; Ruwaard et al., 2009; Spek, Nyklicek et al., 2007; Titov, Andrews, Davies, et al. 2010; van der Zanden et al., 2012; Van Voorhees et al., 2009; Vernmark et al., 2010; Wagner et al., 2014; Warmerdam et al., 2008, 2010) while 10/29 did not use a therapist (Andersson et al., 2005; Bolier et al., 2013; Christensen et al., 2004; Clarke et al., 2009; De Graaf et al., 2009; Lintvedt et al., 2011; Meyer et al., 2009; O'Kearney et al., 2006; Spek et al., 2008; Ünlü Ince et al., 2013) and 2/29 compared therapist- to non-therapist guided CBT (Spek, Nyklicek et al., 2007; Vernmark et al., 2010). *Therapist-guided CBT consistently had a larger effect size across all studies, ranging from 0.6 to 1.9, while stand-alone CBT* (*without therapist guidance*) *had a more modest effect sizes of 0.3–0.7*. Almost all studies showed positive results with there being significant decrease in depression scores; however there were two contrasting results; one RCT where ICBT was found to be inferior to problem-solving therapy (Warmerdam et al., 2008) and the other RCT which did not find any differences between the wait-listed control and the intervention groups (Clarke et al., 2009). In addition, one study on economics-observed Internet-based CBT to be equal in costs (both direct and indirect) when compared to a wait-listed group (Warmerdam et al., 2010).
(b) Other interventions for depression (non-CBT/non-RCT) (Table 3): there were four studies in this section, with varying populations and techniques used (Lipman et al., 2011); (Andersson, Hesser, Hummerdal, Bergman Norgdren, & Carlbring, 2013; Mohr et al., 2010; Van Voorhees, Ellis, Stuart, Fogel, & Ford, 2005). All were open trials and one of these was among adolescents (Van Voorhees et al., 2005). However, modest to high effect sizes [0.2–1.7] were observed at the end of intervention in these studies too.

## Online interventions for anxiety

There were 24 studies that met the inclusion criteria and evaluated Internet interventions for anxiety disorders. 18/24 studies again involved the use of various CBT techniques, including psycho-education, principles of CBT, cognitive restructuring, relaxation, exposure hierarchy and graded exposure, communication and assertiveness skills and relapse prevention. Under the broad rubric of anxiety disorders the following were represented: social anxiety disorders, generalized anxiety disorders (GADs), and mixed anxiety disorders. Panic disorders and phobias were not considered for this review since the nature of interventions differed largely from the other anxiety disorders. Also most studies (*N* = 18) were RCTs, with or without therapist guidance (Andrews, Davies, & Titov, 2011; Berger et al., 2011; Bolier et al., 2013; Carlbring et al., 2011; Carlbring, Nordgren, Furmark, & Andersson, 2009; Hedman, Andersson, Andersson, et al., 2011; Hedman, Andersson, Ljótsson, Andersson, Ruck, et al. 2011; Hedman et al., 2011a; Johansson et al., 2013; Johnston, Titov, Andrews, Spence, & Dear, 2011; Lorian, Titov, & Grisham 2012; Paxling et al. 2011; Robinson et al., 2010; Titov, Andrews, Choi, Schwencke, & Johnston, 2009; Titov, Andrews, Choi, Schwencke, & Johnston, 2009; Titov, Andrews, Choi, Schwencke, & Mahoney, 2008; Titov, Andrews, Johnston, Robinson, & Spence, 2010; Titov, Dear, Schwenke, et al., 2011). Among the other four studies, one was an open trial, one a follow-up study, and two others RCTs of a spiritually integrated treatment technique or a problem-solving intervention technique.

(a) RCTs (Tables 4 and 5): There were 19 RCTs with the majority involving therapist guidance (15/19) (Andrews et al., 2011; Berger et al., 2011; Carlbring et al., 2009, 2011; Hedman, Andersson, Andersson, et al., 2011; Hedman, Andersson, Ljótsson, Andersson, Ruck, et al. 2011; Hedman, Hedman, Andersson, Ljotsson, Andersson, Ruck, & Lindefors, 2011; Johansson et al., 2013, 2011; Paxling et al., 2011; Robinson et al., 2010; Titov et al., 2008, 2011 ; Titov, Andrews, Choi, Schwencke, & Johnston, 2009; Titov, Andrews, Johnston, Robinson, & Spence, 2010), three without therapist guidance (Bolier et al., 2013; Lorian et al., 2012; Titov, Andrews, Choi, Schwencke, & Johnston, 2009) and one compared therapist with non-therapist guidance) (Titov, Gibson, Andrews, & McEvoy, 2009). Since there were different anxiety disorders being reported, there was no single standard measure of scoring. Modules ranged from 6 to 12 weeks in length with follow-up periods ranging from 3 months to 5 years. Most studies on ICBT intervention in anxiety disorders have been on social anxiety, with a few on GAD or mixed groups. *Most studies have also been therapist assisted with robust effect sizes of 0.7–1.7 observed with efficacy similar to face-to-face CBT. Stand-alone CBT studies also were observed to show large effect sizes* (*0.6–1.7*). However, since there were only three non-therapist guided studies, it is difficult to compare them and such conclusions will await further research. All studies showed positive results, and no studies with contrasting results were reported. In addition, one study on the economics of ICBT intervention observed ICBT to be cheaper by about $2000 USD to conventional group-based cognitive therapy (CBGT) in both post-treatment and follow-up costs (Hedman et al., 2011a).
(b) Other interventions for anxiety disorders (non-CBT/non-RCT) (Table 5): There were only four studies in this category (Dear et al. 2011; Hedman, Furmark et al., 2011; Rosmarin, Pargament, Pirutinsky, & Mahoney, 2010; Ünlü Ince et al., 2013), with two being open trials of CBT in social anxiety (Dear et al. 2011; Hedman, Furmark et al., 2011) and the other being an RCT of a spiritually integrated treatment for subclinical anxiety (Rosmarin et al., 2010). Both studies observed significant reductions in anxiety scores at the end of the intervention.

### Methodological quality of included studies

The quality of the included studies was reasonable to good. Prior knowledge of treatment assignment was presented in all studies. In most studies (>85%), outcome measures were self-reported by participants. Drop-out rates varied between 3% and 50%; hence follow-up data also varied.

## Discussion

The findings of this review demonstrate that the Internet is an effective medium for the delivery of interventions designed to reduce the symptoms of depression and anxiety disorders. The effect sizes for both types of conditions were large or at least, modest. In fact, they were at least as large as standard psychological treatment (0.31) in primary care as reported in recent meta-analyses (Titov, Andrews, Choi, Schwencke, & Johnston, 2009). These effect sizes are comparable to the treatment of depression with antidepressant medication (0.37) (Robinson et al., 2010). Similarly, the effect sizes for anxiety interventions reported here are consistent with controlled effect sizes reported for standardized CBT for various anxiety disorders (Hedman et al., 2011c; Johnston et al., 2011; Titov, Andrews, Johnston, Robinson, & Spence, 2010). However, this comes with the caveat that these effect sizes are present regardless of the timing of follow-up assessments, which range from 3 to 36 months.

Most of the studies included here employed an intention-to-treat design. However, recruitment methods varied across studies, as did inclusion criteria. Some studies only included participants with a clinical diagnosis of a depressive or anxiety disorder, while others selected participants on the basis of a clinically significant cut-off score on a self-report measure or questionnaire. Others selected people with elevated but not necessarily clinically significant levels of symptoms, and one study employed a sample of participants with sub-threshold depression, specifically excluding those with a diagnosis of depressive disorder (van der Zanden et al., 2012). Among the studies included, some had assessed effectiveness of oral interventions in clinical samples with diagnosed psychiatric disorders, whereas others had used sub-threshold or only symptomatic diagnosis. A general trend of higher effect size was observed in the clinical, diagnosed samples while a moderate effect size was noted in the group having sub-threshold symptoms.

When it comes to interventions, there are wide variations in delivery as well as in components. Some programs are online versions of self-help manuals with limited or no interaction and others are based more on expert-driven structural frameworks (e.g. Deprexis) and artificial intelligence (AI), tailoring the process based on the experience of the process. When it comes to components of the interventions, there are again wide variations. The techniques that have been used in most depression studies were: cognitive restructuring (used in a total of 13 studies), behavioral activation (8 studies), and psycho-education and relapse prevention (6 studies). It is however important to note that some techniques are referred to differently but are similar to/or are part of, other strategies. For instance, “Thought Diary” and “Challenging Thoughts” are mentioned as individual techniques in one study, but since they are essential components of cognitive restructuring, they were likely used more than evidently shown. On the other hand, some strategies such as assertiveness skills training and sleep management have been used rarely.

With regard to studies about anxiety disorders, graded exposure was a reliable and often used strategy for different anxiety disorders and has been an essential component in 11 studies, while cognitive restructuring and relapse prevention were used in 10 studies each and psycho-education in nine studies, which reflects their importance for the intervention in both depression and anxiety disorders. Comparatively, techniques that were rarely used included self-confrontation and cognitive reappraisal.

In general, the techniques used were reliable and similar to clinical practice, yet some important techniques are used more in real-life CBT than in online interventions; relaxation, for example, has been used in only two studies, probably because it was implied in other techniques such as graded exposure. However, it forms an important component of conventional CBT for anxiety disorders and is one of the techniques used most often, in contrast to its rare use in online interventions. Such variations in the methodology make it impossible to compare across studies and evolve standards for assessment and intervention. Future research therefore needs to focus on effective and standard measures using interactive systems that effectively respond intelligently to the clients.

Based on the current available data, it is not possible to reliably draw conclusions about the factors that predict better outcomes. The effect sizes for anxiety trials appear larger than those for depression trials, but participants in the former trials were more often self-selected volunteers and were typically only included in the trial if they also satisfied diagnostic criteria at screening. There were also high drop-out rates, sometimes reaching 50%, with makes it difficult to comment on the actual effectiveness of the therapies. However, it can also be argued that this reflects treatment settings in the real world, with common drop-out rates reaching 50% in outpatient clinics.

Emerging evidence across trials, especially in depression, clearly suggests that IBIs aim not to replace human interaction, but could help to make much better use of extremely limited expert time. In fact, a direct correlation has been found between the amount of therapist contact in minutes and the between-group effect size (Lorian et al., 2012). However, the picture is not that clear for anxiety disorders, with two studies on CBT without therapist guidance observing near equal effect sizes as those with therapist guidance. Overall, Internet-based treatments are equally efficacious in generating a strong therapeutic alliance (Titov, Andrews, Choi, Schwencke, & Johnston, 2009), while not being strongly associated with outcome. Areas which need to be addressed in the future are the effects of Internet-based treatment related to age and gender, the mechanisms of action and the appropriateness of psychosocial techniques for the online treatment. In addition, it is also essential to explore how these interventions can be integrated into a system of knowledge exchange and assessment strategy.

One of the strengths of this review, which attempted to systematically review the efficacy of online interventions for depression and anxiety, has been the use of original data and outcomes, which allow more clarity and intuitive reading. We believe that transformation of data or outcomes, although important, can sometimes make the review complicated to understand, and we have tried to keep it simple and informative to the reader. In addition, this review has been very exhaustive yet specific, in that it has targeted an understanding of all interventions for anxiety and depression delivered over the Internet. However, the findings of this review have to be understood in light of certain limitations. Despite our broad search, it may be possible that certain good studies may not have been included or been overlooked, due to the search criteria. Also, the desire to be very inclusive may have diluted actual effect sizes or precluded some useful comparisons. Since there were significant differences in study groups, type of interventions and study periods, it was difficult to generate a standardized mean difference (SMD) using pooled data analysis, making any definitive conclusion impossible. Also, we found no trials involving rural residents, older people, or people with low levels of education, which is another limitation of this review. Although we also attempted to analyze the cost-effectiveness of online interventions, the lack of studies prevented us from doing any comprehensive analysis.

## Conclusion

This review documented that the identified programs were successfully delivered in a variety of settings, with differing levels of professional support. This finding highlights the versatility of Internet-based programs and that only brief professional support, if any, is necessary in their delivery. We are led to believe that future interventions would include all groups, especially the rural and the elderly, who would likely also benefit from online interventions.
